# 6,8-Diiodo-5,7-dimeth­oxy-4-methyl­coumarin

**DOI:** 10.1107/S1600536810011360

**Published:** 2010-03-31

**Authors:** P. S. Pereira Silva, Mehtab Parveen, Zakia Khanam, Akhtar Ali, M. Ramos Silva

**Affiliations:** a CEMDRX, Physics Department, University of Coimbra, P-3004-516 Coimbra, Portugal; b Department of Chemistry, Aligarh Muslim University, Aligarh 202 002, India

## Abstract

In the title compound, C_12_H_10_I_2_O_4_, the meth­oxy groups are twisted considerably with respect to the plane of the aromatic ring [CH_3_—O—C—C torsion angles = −85.9 (3) and −92.8 (3)°]. In the crystal, mol­ecules are linked by weak C—H⋯O hydrogen bonds and O⋯I contacts [3.194 (2) Å].

## Related literature

For the medicinal applications of coumarin derivatives, see: Lin *et al.* (2006 ▶); Massimo *et al.* (2003 ▶); Tyagi *et al.* (2003 ▶); Nawrot-Modranka *et al.* (2006 ▶); Sardari *et al.* (1999 ▶); Huang *et al.* (2005 ▶); Elinos-Baez *et al.* (2005 ▶). For the synthesis of the title compound, see: Ali & Ilyas (1986 ▶).
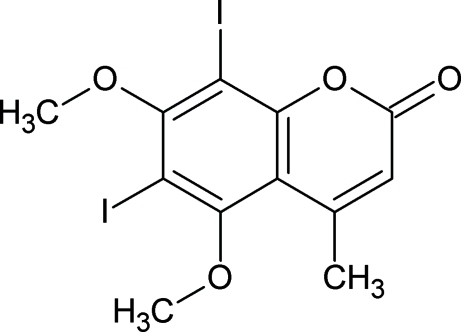


## Experimental

### 

#### Crystal data

C_12_H_10_I_2_O_4_
*M* *_r_* = 472.00
Monoclinic, 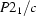

*a* = 10.8681 (2) Å
*b* = 9.1179 (2) Å
*c* = 17.2315 (3) Å
β = 125.395 (1)°
*V* = 1391.95 (5) Å^3^
*Z* = 4
Mo *K*α radiation
μ = 4.52 mm^−1^
*T* = 293 K
0.30 × 0.24 × 0.16 mm

#### Data collection

Bruker APEXII CCD area-detector diffractometer
Absorption correction: multi-scan (*SADABS*; Sheldrick, 2003 ▶) *T*_min_ = 0.396, *T*_max_ = 0.485
27048 measured reflections
3928 independent reflections
3453 reflections with *I* > 2σ(*I*)
*R*_int_ = 0.022

#### Refinement

*R*[*F* ^2^ > 2σ(*F* ^2^)] = 0.023
*wR*(*F* ^2^) = 0.055
*S* = 1.06
3928 reflections
166 parameters
H-atom parameters constrained
Δρ_max_ = 0.71 e Å^−3^
Δρ_min_ = −0.96 e Å^−3^

### 

Data collection: *APEX2* (Bruker, 2003 ▶); cell refinement: *SAINT* (Bruker, 2003 ▶); data reduction: *SAINT*; program(s) used to solve structure: *SHELXS97* (Sheldrick, 2008 ▶); program(s) used to refine structure: *SHELXL97* (Sheldrick, 2008 ▶); molecular graphics: *PLATON* (Spek, 2009 ▶); software used to prepare material for publication: *SHELXL97*.

## Supplementary Material

Crystal structure: contains datablocks global, I. DOI: 10.1107/S1600536810011360/bt5225sup1.cif

Structure factors: contains datablocks I. DOI: 10.1107/S1600536810011360/bt5225Isup2.hkl

Additional supplementary materials:  crystallographic information; 3D view; checkCIF report

## Figures and Tables

**Table 1:** Hydrogen-bond geometry (Å, °)

*D*—H⋯*A*	*D*—H	H⋯*A*	*D*⋯*A*	*D*—H⋯*A*
C3—H3⋯O2^i^	0.93	2.53	3.460 (3)	175

Symmetry code: (i) 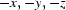.

**Fig. 1.**
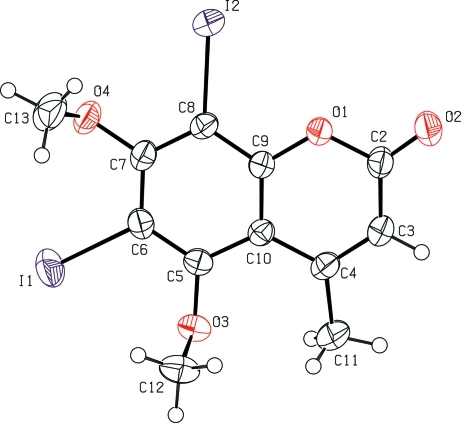
A plot of the title compound. Displacement ellipsoids are drawn at the 50% probability level.

**Fig. 2.**
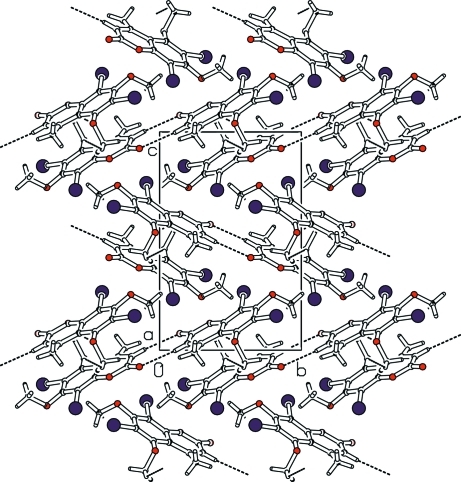
A packing diagram for the title compound, viewed down the a axis, with the hydrogen bonds depicted by dashed lines.

## supplementary crystallographic information

### Comment

Coumarin is the simplest member of the group of oxygen heterocyclics called benzo-2-pyrone. Coumarins are an important class of compounds due to their presence in natural products   as well as their medicinal applications such as anti-inflammatory (Lin *et al.*, 2006), anti-viral (Massimo *et al.*, 2003), antioxidant (Tyagi *et al.*, 2003), antibacterial (Nawrot-Modranka *et al.*, 2006), antifungal (Sardari *et al.*, 1999), anti-HIV (Huang *et al.*, 2005) and as anti-carcinogenic (Elinos-Baez *et al.*, 2005). Besides the wide spectrum of biological applications of coumarin and its derivatives, the chemical literature also embodies their applications as cosmetics, optical brightening agents, and laser dyes. A recent report has revealed the anion sensing ability of some coumarin derivatives. Among various coumarin derivatives, recent pharmacological evaluation of iodocoumarins as cannabinoid receptor antagonists and inverse agonists has been done. Iodocoumarins such as 8-iodo-7-hydroxycoumarin exhibited moderate activity and 8-iodo-5,7-dihydroxycoumarin displayed good antimicrobial properties with MIC values <100 µg/ml. Also, iodocoumarins had been successfully used for the optimization of reaction conditions and kinetic studies in high throughput format. Because of the biological and pharmaceutical importance of iodocoumarins, several protocols for the synthesis have been reported.

In the light of the mentioned above we planned to synthesize iodocoumarins by reaction of 5,7-dimethoxy-4-methylcoumarin with iodine in basic media (Ali & Ilyas, 1986).

In the molecule of the title compound (Fig. 1), the best plane through the aromatic ring shows an r.m.s. deviation of 0.0154 Å; the O1—C2—C3—C4—C10—C9 ring shows a slightly larger deviation from planarity, with an r.m.s. deviation of 0.0189 Å. The angle between these two planes is 4.96 (11)°.

The C2 atom of the carbonyl group has a distorted trigonal geometry with O2—C2—O1 [116.6 (2)°] and O2—C2—C3 [126.5 (2)°] deviating significantly from the ideal sp^2^ value of 120°.

The methoxy groups are considerably twisted with respect to the plane of the aromatic ring as indicated by the torsion angles C12—O3—C5—C6 [-85.9 (3)°] and C13—O4—C7—C6 [90.7 (3)°]. The iodine atoms are almost in the plane of the benzene ring and the methyl group is slightly out of the pyrone ring plane.

The molecules  are linked across an inversion centre by one weak hydrogen bond of the C—H···O type (Fig. 2, Table 2).

### Experimental

To a stirred solution of 5,7-dimethoxy-4-methylcoumarin (2.20 g, 10 mmol) in 15-20 ml of methanol containing 8.2 g KOH was dropwise added to a solution of I_2_ (2.56 g, 10 mmol) over a period of 30 min and stirred at room temperature for about 2 hours. The reaction mixture was poured into water and residual iodine was removed by washing with sodium thiosulphate. On treatment with sodium thiosulphate we obtained a precipitate which was filtered and crystallized with CHCl_3_—MeOH as white crystals (300 mg, m.p. 490 K).

### Refinement

All H atoms were located in a difference Fourier synthesis, placed in calculated positions and refined as riding on their parent atoms, using SHELXL97 (Sheldrick, 2008) defaults.

### Crystal data

**Table:**

C_12_H_10_I_2_O_4_	*F*(000) = 880
*M**_r_* = 472.00	*D*_x_ = 2.252 Mg m^−^^3^
Monoclinic, *P*2_1_/*c*	Melting point: 490 K
Hall symbol: -P 2ybc	Mo *K*α radiation, λ = 0.71073 Å
*a* = 10.8681 (2) Å	Cell parameters from 7934 reflections
*b* = 9.1179 (2) Å	θ = 2.4–29.5°
*c* = 17.2315 (3) Å	µ = 4.52 mm^−^^1^
β = 125.395 (1)°	*T* = 293 K
*V* = 1391.95 (5) Å^3^	Irregular block, pale yellow
*Z* = 4	0.30 × 0.24 × 0.16 mm

### Data collection

**Table:**

Bruker APEXII CCD area-detector diffractometer	3928 independent reflections
Radiation source: fine-focus sealed tube	3453 reflections with *I* > 2σ(*I*)
graphite	*R*_int_ = 0.022
φ and ω scans	θ_max_ = 29.7°, θ_min_ = 2.3°
Absorption correction: multi-scan (*SADABS*; Sheldrick, 2003)	*h* = −14→15
*T*_min_ = 0.396, *T*_max_ = 0.485	*k* = −12→12
27048 measured reflections	*l* = −24→23

### Refinement

**Table:**

Refinement on *F*^2^	Primary atom site location: structure-invariant direct methods
Least-squares matrix: full	Secondary atom site location: difference Fourier map
*R*[*F*^2^ > 2σ(*F*^2^)] = 0.023	Hydrogen site location: inferred from neighbouring sites
*wR*(*F*^2^) = 0.055	H-atom parameters constrained
*S* = 1.06	*w* = 1/[σ^2^(*F*_o_^2^) + (0.0226*P*)^2^ + 1.1738*P*] where *P* = (*F*_o_^2^ + 2*F*_c_^2^)/3
3928 reflections	(Δ/σ)_max_ = 0.001
166 parameters	Δρ_max_ = 0.71 e Å^−^^3^
0 restraints	Δρ_min_ = −0.96 e Å^−^^3^

### Special details

**Table:**

Geometry. All esds (except the esd in the dihedral angle between two l.s. planes) are estimated using the full covariance matrix. The cell esds are taken into account individually in the estimation of esds in distances, angles and torsion angles; correlations between esds in cell parameters are only used when they are defined by crystal symmetry. An approximate (isotropic) treatment of cell esds is used for estimating esds involving l.s. planes.
Refinement. Refinement of *F*^2^ against ALL reflections. The weighted *R*-factor wR and goodness of fit *S* are based on *F*^2^, conventional *R*-factors *R* are based on *F*, with *F* set to zero for negative *F*^2^. The threshold expression of *F*^2^ > σ(*F*^2^) is used only for calculating *R*-factors(gt) etc. and is not relevant to the choice of reflections for refinement. *R*-factors based on *F*^2^ are statistically about twice as large as those based on *F*, and *R*- factors based on ALL data will be even larger.

### Fractional atomic coordinates and isotropic or equivalent isotropic displacement parameters (Å^2^)

**Table:**

	*x*	*y*	*z*	*U*_iso_*/*U*_eq_	
I1	0.51053 (2)	0.82363 (2)	0.156895 (15)	0.05749 (7)	
I2	0.11277 (2)	0.593603 (19)	0.266073 (13)	0.04546 (6)	
O1	0.06827 (19)	0.35475 (17)	0.12276 (12)	0.0353 (3)	
O2	−0.0507 (2)	0.1432 (2)	0.07590 (17)	0.0548 (5)	
O3	0.39384 (18)	0.53122 (19)	0.03873 (12)	0.0383 (4)	
O4	0.3379 (2)	0.79647 (18)	0.25451 (12)	0.0434 (4)	
C8	0.1966 (3)	0.5766 (2)	0.18397 (16)	0.0317 (4)	
C7	0.2959 (3)	0.6823 (2)	0.19268 (16)	0.0337 (5)	
C6	0.3586 (3)	0.6668 (2)	0.14197 (17)	0.0352 (5)	
C5	0.3273 (2)	0.5453 (3)	0.08510 (15)	0.0318 (4)	
C10	0.2315 (2)	0.4324 (2)	0.07795 (15)	0.0297 (4)	
C9	0.1647 (2)	0.4549 (2)	0.12598 (15)	0.0294 (4)	
C4	0.2056 (3)	0.2920 (2)	0.03026 (16)	0.0352 (5)	
C3	0.1095 (3)	0.1972 (3)	0.02911 (18)	0.0395 (5)	
H3	0.0905	0.1087	−0.0028	0.047*	
C2	0.0351 (3)	0.2248 (3)	0.07400 (18)	0.0379 (5)	
C13	0.2462 (4)	0.9256 (3)	0.2126 (2)	0.0621 (8)	
H13A	0.2393	0.9513	0.1562	0.093*	
H13B	0.2911	1.0051	0.2573	0.093*	
H13C	0.1470	0.9066	0.1964	0.093*	
C12	0.3139 (3)	0.6047 (3)	−0.05204 (19)	0.0497 (6)	
H12A	0.2153	0.5623	−0.0936	0.075*	
H12B	0.3687	0.5937	−0.0797	0.075*	
H12C	0.3044	0.7070	−0.0433	0.075*	
C11	0.2873 (4)	0.2436 (3)	−0.0117 (2)	0.0582 (8)	
H11A	0.2607	0.1440	−0.0331	0.087*	
H11B	0.3942	0.2502	0.0357	0.087*	
H11C	0.2592	0.3057	−0.0646	0.087*	

### Atomic displacement parameters (Å^2^)

**Table:**

	*U*^11^	*U*^22^	*U*^33^	*U*^12^	*U*^13^	*U*^23^
I1	0.05881 (12)	0.05243 (11)	0.06135 (13)	−0.02587 (9)	0.03486 (10)	−0.01030 (8)
I2	0.05746 (11)	0.04523 (10)	0.04782 (10)	−0.00537 (7)	0.03859 (9)	−0.01056 (7)
O1	0.0400 (9)	0.0303 (7)	0.0438 (9)	−0.0055 (6)	0.0290 (8)	−0.0069 (7)
O2	0.0650 (13)	0.0391 (9)	0.0823 (15)	−0.0165 (9)	0.0552 (12)	−0.0145 (10)
O3	0.0370 (8)	0.0458 (9)	0.0384 (9)	0.0023 (7)	0.0255 (8)	0.0035 (7)
O4	0.0572 (11)	0.0325 (8)	0.0355 (9)	−0.0062 (8)	0.0239 (8)	−0.0092 (7)
C8	0.0355 (11)	0.0304 (10)	0.0307 (10)	0.0031 (8)	0.0200 (9)	−0.0015 (8)
C7	0.0380 (12)	0.0277 (10)	0.0286 (10)	−0.0006 (8)	0.0154 (9)	−0.0023 (8)
C6	0.0355 (11)	0.0325 (11)	0.0330 (11)	−0.0057 (9)	0.0172 (10)	0.0007 (9)
C5	0.0299 (10)	0.0357 (11)	0.0291 (10)	0.0019 (8)	0.0167 (9)	0.0032 (8)
C10	0.0307 (10)	0.0294 (10)	0.0271 (10)	0.0008 (8)	0.0157 (9)	−0.0008 (8)
C9	0.0293 (10)	0.0280 (9)	0.0296 (10)	0.0003 (8)	0.0162 (9)	−0.0005 (8)
C4	0.0400 (12)	0.0334 (11)	0.0338 (11)	0.0017 (9)	0.0224 (10)	−0.0036 (9)
C3	0.0477 (14)	0.0299 (11)	0.0453 (13)	−0.0041 (10)	0.0294 (12)	−0.0077 (9)
C2	0.0409 (12)	0.0290 (10)	0.0453 (13)	−0.0031 (9)	0.0257 (11)	−0.0049 (9)
C13	0.093 (2)	0.0340 (13)	0.0625 (19)	0.0072 (15)	0.0471 (19)	−0.0025 (12)
C12	0.0480 (15)	0.0696 (18)	0.0379 (13)	0.0039 (13)	0.0285 (12)	0.0077 (12)
C11	0.078 (2)	0.0493 (16)	0.077 (2)	−0.0089 (15)	0.0618 (19)	−0.0206 (15)

### Geometric parameters (Å, °)

**Table:**

I1—C6	2.084 (2)	C10—C4	1.458 (3)
I2—C8	2.085 (2)	C4—C3	1.347 (3)
O1—C9	1.367 (3)	C4—C11	1.500 (3)
O1—C2	1.375 (3)	C3—C2	1.428 (3)
O2—C2	1.208 (3)	C3—H3	0.9300
O3—C5	1.359 (3)	C13—H13A	0.9600
O3—C12	1.441 (3)	C13—H13B	0.9600
O4—C7	1.365 (3)	C13—H13C	0.9600
O4—C13	1.438 (3)	C12—H12A	0.9600
C8—C7	1.389 (3)	C12—H12B	0.9600
C8—C9	1.396 (3)	C12—H12C	0.9600
C7—C6	1.392 (3)	C11—H11A	0.9600
C6—C5	1.385 (3)	C11—H11B	0.9600
C5—C10	1.418 (3)	C11—H11C	0.9600
C10—C9	1.397 (3)		
			
C9—O1—C2	121.60 (18)	C4—C3—C2	123.8 (2)
C5—O3—C12	113.74 (18)	C4—C3—H3	118.1
C7—O4—C13	114.3 (2)	C2—C3—H3	118.1
C7—C8—C9	118.9 (2)	O2—C2—O1	116.6 (2)
C7—C8—I2	119.53 (16)	O2—C2—C3	126.5 (2)
C9—C8—I2	121.34 (16)	O1—C2—C3	116.9 (2)
O4—C7—C8	120.0 (2)	O4—C13—H13A	109.5
O4—C7—C6	120.2 (2)	O4—C13—H13B	109.5
C8—C7—C6	119.6 (2)	H13A—C13—H13B	109.5
C5—C6—C7	121.1 (2)	O4—C13—H13C	109.5
C5—C6—I1	119.26 (17)	H13A—C13—H13C	109.5
C7—C6—I1	119.58 (16)	H13B—C13—H13C	109.5
O3—C5—C6	119.7 (2)	O3—C12—H12A	109.5
O3—C5—C10	119.6 (2)	O3—C12—H12B	109.5
C6—C5—C10	120.6 (2)	H12A—C12—H12B	109.5
C9—C10—C5	116.74 (19)	O3—C12—H12C	109.5
C9—C10—C4	117.8 (2)	H12A—C12—H12C	109.5
C5—C10—C4	125.4 (2)	H12B—C12—H12C	109.5
O1—C9—C10	121.85 (19)	C4—C11—H11A	109.5
O1—C9—C8	115.25 (19)	C4—C11—H11B	109.5
C10—C9—C8	122.8 (2)	H11A—C11—H11B	109.5
C3—C4—C10	117.8 (2)	C4—C11—H11C	109.5
C3—C4—C11	118.3 (2)	H11A—C11—H11C	109.5
C10—C4—C11	123.7 (2)	H11B—C11—H11C	109.5
			
C13—O4—C7—C8	−92.8 (3)	C2—O1—C9—C10	1.7 (3)
C13—O4—C7—C6	90.7 (3)	C2—O1—C9—C8	−175.3 (2)
C9—C8—C7—O4	−175.0 (2)	C5—C10—C9—O1	178.85 (19)
I2—C8—C7—O4	−0.1 (3)	C4—C10—C9—O1	−5.2 (3)
C9—C8—C7—C6	1.5 (3)	C5—C10—C9—C8	−4.3 (3)
I2—C8—C7—C6	176.40 (17)	C4—C10—C9—C8	171.7 (2)
O4—C7—C6—C5	174.5 (2)	C7—C8—C9—O1	178.8 (2)
C8—C7—C6—C5	−2.0 (3)	I2—C8—C9—O1	4.0 (3)
O4—C7—C6—I1	−2.4 (3)	C7—C8—C9—C10	1.7 (3)
C8—C7—C6—I1	−178.96 (17)	I2—C8—C9—C10	−173.05 (16)
C12—O3—C5—C6	−85.9 (3)	C9—C10—C4—C3	5.1 (3)
C12—O3—C5—C10	96.7 (3)	C5—C10—C4—C3	−179.3 (2)
C7—C6—C5—O3	−178.0 (2)	C9—C10—C4—C11	−170.8 (3)
I1—C6—C5—O3	−1.1 (3)	C5—C10—C4—C11	4.8 (4)
C7—C6—C5—C10	−0.7 (3)	C10—C4—C3—C2	−1.8 (4)
I1—C6—C5—C10	176.25 (16)	C11—C4—C3—C2	174.3 (3)
O3—C5—C10—C9	−178.92 (19)	C9—O1—C2—O2	179.6 (2)
C6—C5—C10—C9	3.7 (3)	C9—O1—C2—C3	1.7 (3)
O3—C5—C10—C4	5.4 (3)	C4—C3—C2—O2	−179.2 (3)
C6—C5—C10—C4	−171.9 (2)	C4—C3—C2—O1	−1.7 (4)

### Hydrogen-bond geometry (Å, °)

**Table:**

*D*—H···*A*	*D*—H	H···*A*	*D*···*A*	*D*—H···*A*
C3—H3···O2^i^	0.93	2.53	3.460 (3)	175

Symmetry codes: (i) −*x*, −*y*, −*z*.

## References

[bb1] Ali, S. M. & Ilyas, M. (1986). *J. Org. Chem.* **51**, 5415–5417.

[bb2] Bruker (2003). *APEX2* and *SAINT* Bruker AXS Inc., Madison, Wisconsin, USA.

[bb3] Elinos-Baez, C. M., Leon, F. & Santos, E. (2005). *Cell Biol. Int.* **29**, 703–708. 10.1016/j.cellbi.2005.04.003 15964220

[bb4] Huang, L., Yuon, X., Yu, D., Lee, K. H. & Chin, H. C. (2005). *Virology*, **332**, 623–628. 10.1016/j.virol.2004.11.033 15680427

[bb5] Lin, C. M., Huang, S. T., Lee, F. W., Sawkuo, H. & Lin, M. H. (2006). *Bioorg. Med. Chem.* **14**, 4402–4409. 10.1016/j.bmc.2006.02.042 16540334

[bb6] Massimo, C., Francesco, E., Federica, M., Carla, M. M., Prieto, G. S. & Carlos, R. J. (2003). *Aust. J. Chem.* **56**, 59–60.

[bb7] Nawrot-Modranka, J., Nawrot, E. & Graczyk, J. (2006). *Eur. J. Med. Chem.* **41**, 1301–1309. 10.1016/j.ejmech.2006.06.004 16904795

[bb8] Sardari, S., Mori, Y., Horita, K., Micetich, R. G., Nishibe, S. & Daneshtalab, M. (1999). *Bioorg. Med. Chem.* **7**, 1933–1940. 10.1016/s0968-0896(99)00138-8 10530942

[bb9] Sheldrick, G. M. (2003). *SADABS* University of Göttingen, Germany.

[bb10] Sheldrick, G. M. (2008). *Acta Cryst.* A**64**, 112–122. 10.1107/S0108767307043930 18156677

[bb11] Spek, A. L. (2009). *Acta Cryst.* D**65**, 148–155. 10.1107/S090744490804362X PMC2631630 19171970

[bb12] Tyagi, A. K., Raj, H. G., Vohra, P., Gupta, G., Kumari, R., Kumar, P. & Gupta, R. K. (2003). *Eur. J. Med. Chem.* **40**, 413–420. 10.1016/j.ejmech.2004.09.002 15804541

